# Supplementary material for: Polygenic Risk of Hypertriglyceridemia Is Modified by BMI
Source: Int J Mol Sci. 2022 Aug 30;23(17):9837. doi: 10.3390/ijms23179837 (PMC9456481; doi:10.3390/ijms23179837)
Supplement: Supplementary file 1 [file ijms-23-09837-s001.zip › ijms-1866221-supplementary.pdf]

Table S1. Drugs excluded from the study for causing hypertriglyceridemia

	Active pharmaceutical ingredient
Hormone therapy	Estrogen, 2n, 3rd generation progestogens, tamoxifen, steroids
Retinoids	Isotretioin, acitretin
Immunossupresors	Cyclosporine, azathioprine, sirolimus
Protease inhibitors	Amprenavir, indinavir, nelfinavir, ritonavir, saquinavir
Antipshychotic drugs	clozapine
Bile acid sequestrants	Cholestyramine, colestipol, colesvelam

Information extracted from Catalan Institute of Pharmacology

Table S2: Description of 6 allelic variants

Gene	SNP (rs)	genomic coordinates	Change sequency	coding/protein-coding notation	Zone	Risk allele	Allele frequency (GNOMAD)	minor allele frequency (MAF)
ZPR1	Rs964184	NC_000011.10:g.116778201G>C	NM_003904.4: c.*724C>G	NP	3'UTR	G	G=0.14838<br>C=0.85162	G=0.14838
APOA5	Rs3135506	NC_000011.10:g.116791691G>C	NM_052968.4: c.56C>G	NP_00160070.1: p.Ser19Leu	exonic	G	G=0.0557<br>C=0.9443	G=0.0557
GCKR	Rs1260326	NC_000002.12:g.27508073T>G	NM_001486.3:c.133T>C	NP_001477.2:p.Leu446Pro	exonic	T	C=0.67473<br>T=0.32527	T=0.32527
LPL	Rs12678919	NC_000008.11:g.19986711	NC_000008.11:g.19986711A>G	NP	intergenic	A	A=0.90331<br>G=0.09669	G=0.09669
BAZ1B	Rs7811265	NC_000007.14:g.73520180A>G	NM_032408.3:c.107+1647T>C	NP	intronic	A	A=0.77928<br>G=0.22072	G=0.22072
TRIB	Rs2954029	NC_000008.11:g.125478730	NC_000008.11:g.125478730A>T	NP	intergenic	A	A=0.56755<br>T=0.43245	T=0,43245

Table S3. Baseline characteristics of the study participants by gender.

CLINICAL VARIABLES	Total n=276	gender Male n=204	Female N=72	p
Age (years)	52.1 (10.5)	50.4 (10.2)	56.7 (10.0)	<0.001
BMI (kg/m^2)	28.7 (3.8)	28.9 (3.7)	28.0 (4.2)	0.065
Waist circumference (cm)	100.4 (12.5)	102.2 (11.4)	95.7 (14.1)	0.002
Current or former-smoker (%)	169 (61.2%)	134 (65.7%)	35 (48.6%)	0.011
Alcohol consumption (units/w)	1.2 (2.7)	1.4 (2.9)	0.4 (1.8)	0.009
Diet score (*)	7.5 (3.3)	7.3 (3.4)	7.6 (3.3)	0.440
Physical activity (hours/week)	2.6 (3.0)	2.5 (3.2)	2.7 (2.9)	0.558
HTA (%)	94 (34.1%)	65 (31.9%)	29 (40.3%)	0.195
DM (%)	49 (17.8)	37 (18.1%)	12 (16.7%)	0.779
Chronic renal disease (%)	19 (6.9)	14 (6.9%)	5 (6.9%)	0.981
Atherothrombotic cardiovascular disease	75 (27.2%)	55 (27.0%)	20 (27.8%)	0.893
Pancreatitis (%)	2 (0.7%)	2 (1.0%)	0 (0%)	>0.999
Total cholesterol (mmol/L)*	6.7 (1.8)	6.6 (1.7)	7.1 (1.9)	0.040
HDL-c (mmol/L)*	0.98 (0.32)	0.92 (0.26)	1.13 (0.4)	<0.001
LDL-c (mmol/L)*	3.6 (1.5)	3.5 (1.6)	3.8 (1.4)	0.347

**non-HDL-c (mmol/L)***	5.7 (1.8)	5.7 (1.8)	6.0 (1.9)	0.236
**TG (mmol/)**	5.3 (3.4 to 9.6)	5.4 (3.4 to 9.9)	4.5 (3.2 to 8.6)	0.173
**ApoB (mg/dL)**	1.2 (0.4)	1.2 (0.4)	1.2 (0.4)	0.440
**HbA1c (%)**	5.87 (0.71)	5.89 (0.77)	5.80 (0.47)	0.377

*Results are expressed as mean and standard deviation (sd), median (interquartile range) or in n (percentage). * Diet score was calculated according to a standardized questionnaire [18]. GRS: Genetic Risk Score; DM: diabetes mellitus; ApoB: apolipoprotein B; HbA1c: glycated hemoglobin; BMI: body mass index; HTA: hypertension; TC: total cholesterol, LDL-c: low density lipoprotein cholesterol, HDL-c: high density lipoprotein cholesterol, non-HDL-c: non-high density lipoprotein cholesterol, TG: triglycerides. TC, HDL-c, LDL-c, non-HDL-c values correspond to the blood test with the highest TG values for each patient.*

Table S4: Comparison of the frequency of the allelic variants frequency observed between the general population and our sample.

GENE (VARIANT)	WILD TYPE	HETEROZYGOUS	HOMOZYGOUS	P
**ZPR1 (rs964184)**	**CC**	**CG**	**GG**	2.089×10^{-34}
**General**	77%	22%	1%	
**Study sample**	130 (47.1%)	120 (43.5%)	26 (9.4%)	
**APOA5 (rs3135506)**	**CC**	**CG**	**GG**	5.673×10^{-57}
**General**	94%	6%	0.001%	
**Study sample**	199 (72.1%)	68 (24.6%)	9 (3.3%)	
**GCKR (rs1260326)**	**CC**	**CT**	**TT**	6.913×10^{-07}
**General**	33%	50%	17%	
**Study sample**	54 (19.6%)	153 (55.4%)	69 (25.0%)	
**LPL (rs12678919)**	**GG**	**AG**	**AA**	0.00043
**General**	3.5%	16%	80.5%	
**Study sample**	2 (0.7%)	29 (10.5%)	245 (88.8%)	
**BAZ1B (rs78112659)**	**CC**	**CT**	**TT**	4.991×10^{-10}
**General**	3.5%	34.5%	62%	
**Study sample**	9 (3.3%)	47 (17.0%)	220 (79.7%)	
**TRIB (rs29540299)**	**TT**	**TA**	**AA**	0.00193
**General**	10%	57%	33%	
**Study sample**	28 (10.1%)	130 (47.1%)	118 (42.8%)	

Population frequency data have been obtained from 1000 genome datasets (available in https://www.internationalgenome.org/)

Table S5: Analysis of the interaction between the genetic risk score (GRS) and different clinical variables

Model		Unstandardized Coefficients	95% Confidence Interval Lower Bound	95% Confidence Interval Upper Bound	Standardized Coefficients	Sig.
BMI	(Constant)	22,890	-2,749	48,528		,080
	GRS	-2,963	-6,677	,751	-,667	,117
	BMI (kg/m2)	-,847	-1,745	,052	-,487	,065
	GRS*BMI interaction	,149	,019	,278	1,163	**,024**
Gender	(Constant)	-1,980	-6,367	2,407		,375
	GRS	1,463	,837	2,088	,324	,000
	Gender	3,753	-4,379	11,886	,245	,364
	GRS*Gender interaction	-,708	-1,829	,413	-,343	,215
Age	(Constant)	-8,657	-27,637	10,323		,370
	GRS	2,477	-,120	5,075	,548	,061
	Age	,156	-,204	,515	,242	,394
	GRS*Age interaction	-,024	-,073	,025	-,400	,329
Diet score	(Constant)	1,184	-8,125	10,493		,802
	GRS	1,101	-,229	2,432	,243	,104
	Diet score	-,223	-1,351	,905	-,110	,698
	GRS*Diet interaction	,013	-,147	,174	,051	,872
DM	(Constant)	-,502	-4,500	3,495		,805
	GRS	1,151	,591	1,712	,255	,000
	DM	-1,315	-11,446	8,815	-,075	,798
	GRS*DM interaction	,471	-,973	1,915	,188	,521
Physical activity	(Constant)	-1,486	-6,578	3,606		,566
	GRS	1,426	,702	2,151	,310	,000
	Physical activity (h/w)	-,007	-1,298	1,284	-,003	,991
	GRS*Physic.Act. interac.	-,023	-,200	,153	-,079	,793
Alcohol consumption	(Constant)	,051	-4,059	4,160		,981
	GRS	1,141	,558	1,724	,252	,000
	Alcohol cons.(u/w)	-,829	-2,316	,658	-,331	,273
	GRS*Alcohol interaction	,115	-,114	,344	,297	,324

DM: Diabetes mellitus. Physic.Act. interact: Physical activity interaction.
